# Cell-derived biomimetic nanoparticles as a novel drug delivery system for atherosclerosis: predecessors and perspectives

**DOI:** 10.1093/rb/rbaa019

**Published:** 2020-05-25

**Authors:** Long Yang, Guangchao Zang, Jingwen Li, Xinyue Li, Yuanzhu Li, Yinping Zhao

**Affiliations:** Laboratory of Tissue and Cell Biology, Lab Teaching & Management Center, Chongqing Medical University, NO.1 Medical College Road, Yuzhong District, Chongqing 400016, China

**Keywords:** atherosclerosis, nanoparticle, cell membrane, extracellular vesicle

## Abstract

Atherosclerosis is a key mechanism underlying the pathogenesis of cardiovascular disease, which is associated with high morbidity and mortality. In the field of precision medicine for the treatment of atherosclerosis, nanoparticle (NP)-mediated drug delivery systems have great potential, owing to their ability to release treatment locally. Cell-derived biomimetic NPs have attracted extensive attention at present due to their excellent targeting to atherosclerotic inflammatory sites, low immunogenicity and long blood circulation time. Here, we review the utility of cell-derived biomimetic NPs, including whole cells, cell membranes and extracellular vesicles, in the treatment of atherosclerosis.

## Introduction

Atherosclerosis often leads to life-threatening cardiovascular diseases such as myocardial infarction and stroke [1]. It has become one of the most common clinical diseases around the world and poses a serious threat to the health of the human population [2]. The pathogenesis of atherosclerosis is complex and diverse, often involving changes in, and interactions between, various cell types in the arterial wall [3, 4]. The pathogenesis also involves the accumulation of lipids, cholesterol and fibrous elements in the innermost layer of medium- and large-sized arterial intima [5, 6].

Traditionally, stenting and nanoparticle (NP) technology are used to treat atherosclerosis [7]. However, both the drug-eluting stent and the bare metal stent cause restenosis and stent thrombosis to a certain extent [8]. Much of the NP technology that might be used for drug delivery utilize organic, inorganic or metallic materials that show cytotoxicity and low biocompatibility-features that need to be overcome in order for this technology to be used in the treatment of atherosclerosis. As nanotechnology improves, there is an ongoing effort to create NPs coated with natural cells that can be used as drug carriers to overcome the above limitations [9].

Recent studies ascertain that biomimetic NPs can achieve long-term blood circulation [10], more effective immune evasion and better targeting compared to traditional NPs [11]. Moreover, biomimetic NPs are more likely to be considered as ‘self’, thus demonstrating improved biocompatibility versus traditional NPs [12]. In addition, these cell-derived drug delivery systems can successfully transfer phospholipid bilayers from natural cells to nanocarriers without damaging functional surface proteins [13]. The application of biomimetic NP technology in the treatment of cardiovascular diseases has gradually increased, and the most-used technologies can be divided into three types: whole cell, cell membrane and vesicles-based NPs [14].

In this review, we discuss the recent progress in the use of whole cells, cell membranes and extracellular vesicle (EV)-camouflaged NPs drug delivery systems for the treatment of atherosclerosis (Table 1). Specifically, we describe the composition, characteristics, applications and future prospects of the biomimetic NP technology in this therapeutic field.

**Table 1:** The compositions, characteristics, applications and prospects of cell-derived biomimetic drug delivery systems

Construction	Classification	Advantage	Disadvantage	Functional principle	Application and prospect	Ref.
Red blood cells (RBC)	Directly encapsulate NPs into RBC. Bound drug or NPs to RBCm surface. RBCm coated NPs.	Easily collected. Long drug circulation. Sustained drug release. Non-immunogenicity. Restrict unintended extravasation out of vessel. Highly biocompatibility.	Restricted to the bloodstream. Lack of targeting capability. Short storage time. Environmental stimuli make the particles detached from RBCs quickly.	Membrane protein CD47 and CD59 prolong circulation time and provide immune escape ability. Enhanced pharmacokinetic profiles due to the membrane components such as hydrophilic sialic acid. Passive accumulation in solid tumours due to the EPR effect.	Fusion with other membranes. Senescent RBCs membrane coated NPs can promote phagocytosis of antigen presenting cells (APCs) which are used as nanovaccine or target to the spleen and liver.	[15–25]
Platelet	Loading drug. Platelet membrane coated NPs.	Good biocompatibility. Little macrophage phagocytosis. Long circulation time. Homing to injured vascular and tumour cells. Nice carriers for anti-thrombotic drug delivery.	Promoted thrombosis and tumourigenesis. Limited lifetime. Not enough therapeutic stability. Lack of mass production. Immunogenicity.	Platelet activation come with dynamic morphology and functions change which are exploited to trigger drug release. The surface molecules on platelet membrane such as P-selectin and CD40 ligand modulate disease process in inflammation.	Shear-sensitive anti-thrombotic therapy has promising future owing to the shear stress-dependent interaction of platelet receptor GPIBα with the A1 domain of VWF. Aim at circulating tumour cell. Synthesis of hybrid membrane.	[26–30]
Macrophage	MI macrophage. MII macrophage.	Actively target the damaged endothelium region and inflammation. Improved treatment and reduced side effects. Long circulation time.	Short storage time. Prone to switch to the proinflammatory phenotype. High heterogeneity. Lack of mass and efficient production.	With numerous adhesive receptors and homing ability toward cytokines released from diseased tissue. Surface functional proteins such as integrin-α4β1, specifically recognize VCAM-1.	Bacteria pre-treatment to improve the anti- bacterial ability of the camouflaged system. Synthesis of hybrid membrane. Great potential for drug delivery to hypoxic tumour niches.	[31–36]
Extracellular vesicles (EVs)	Microvesicles, apoptotic bodies, exosomes. Erythrocyte, platelet, leukocyte and endothelial EVs.	Nano-size. Increased cell internalization efficiency. Long circulation time.	Little is known about the physiological role of EVs. Worse side effect. Hard to isolate and no standard preservation method.	Good EPR effect. Biological properties derive from their sophisticate membrane structure and protein for active targeting, adhesion, cell fusion and drug release.	Developed as biomarkers in cardiovascular disease. Serve as therapeutic agents in many fields.	[37–44]

## Pathogenesis of atherosclerosis

**Figure 1:**
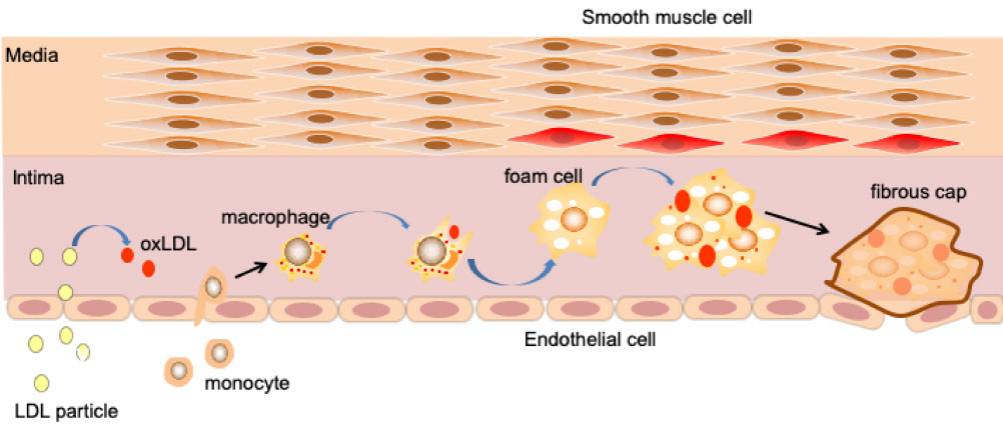
Schematic diagram of vascular cavity rupture process of atherosclerotic plaque formation. When endothelial cells (ECs) are damaged or affected by other factors, they will increase the permeability to low-density lipoprotein (LDL). After entering the intimal layer, LDL particles are transformed into oxidized low-density lipoprotein (ox-LDL), which stimulate activated ECs to release inflammatory factors that recruit monocytes. These monocytes differentiate into macrophages and phagocytose ox-LDL, leading to the formation of foam cells and atherosclerotic plaques with a necrotic lipid core. Finally, the fibrous cap forms and covers the necrotic lipid core plaque, which upon rupture will enter the lumen together.

The conception of atherosclerosis comes from the Greek words ‘athera’, meaning ‘porridge’, and ‘sclereni’, meaning ‘hardening’. The disease manifests as an accumulation of a thick, porridge-like yellow lipid on the inner wall of blood vessels [45, 46]. The initial event in the pathogenesis of atherosclerosis is endothelial dysfunction [47], which promotes the accumulation of low-density lipoprotein (LDL) in the arterial intima. This is followed by transformation of LDL to oxidized LDL (ox-LDL) in the subendothelial layer [48] (Fig. 1).

Ox-LDL particles contribute to endothelial cell (EC) activation and dysfunction, smooth muscle cell (SMC) migration and proliferation, and platelet activation:

- Activated ECs not only release chemokines and proinflammatory factors, but highly express adhesion molecules (such as vascular cell adhesion molecule-1 [VCAM-1], intercellular adhesion molecule-1 [ICAM-1] and growth factors, e.g. macrophage colony stimulating factor) that interact with monocyte receptors to stimulate homing and migration to damaged areas and differentiation into macrophages [49, 50].
- In advanced atherosclerosis, numerous SMCs migrate from the vascular media to intima, resulting in arterial stenosis.
- Activated platelets are attached to the damaged vessel wall through the GPIIb/IIIa [51], ICAM-1 or vitronectin receptor [52]. They not only directly participate in thrombotic events but also release pro-inflammatory mediators that can recruit leukocytes [53].

Macrophages adhere to damaged blood vessels through specific recognition of integrin α4β1 and VCAM-1 on the membrane. Then the recruited macrophages phagocytize ox-LDL [54] via scavenger receptors and transform into foam cells [55]. These cells accumulate in a plaque and undergo apoptosis, forming a lipid-rich necrotic core. Macrophages also secrete growth factors that promote the production of collagen and the deposition of extracellular matrix [55], which is followed by the formation of fibrous caps under the endothelium [56] (Fig. 1). Moreover, macrophages secrete pro-inflammatory cytokines and encourage the modification of matrix metalloproteinases (MMPs) [57, 58]. Presence of MMPs is thought to be the main cause of the thinning and rupture of fibrous caps, an event followed by release of the thrombotic necrotic core into the blood [59] and a pathological process that can result in thrombosis [60, 61].

In summary, atherosclerosis is a chronic inflammatory disease involving the accumulation of lipid, activation of pro-inflammatory signalling pathways, cytokine/chemokine release from recruited cells and increased oxidative stress.

## Whole cells as drug carriers

Drug delivery systems based on nanotechnology have been developed in many biomedical fields [62]. In the early 1970s, scientists noticed that the red blood cell (RBC) membrane, which is semi permeable, could protect the enzymes encapsulated in it [16]. This led to the first clinical trial of a drug delivery system based on intact RBCs [63]. Following this discovery, the diversity of cells used as nano-carriers has continued to increase, and now the combination of active and passive targeting nanomaterials is known to significantly improve drug absorption rate at disease sites [64, 65].

In general, whole-cell drug delivery systems have the following advantages: (i) they can prevent drug degradation and inactivation; (ii) they can pass through the physiological barrier of human body; (iii) they have a longer blood circulation time than NPs; (iv) they have a high drug loading capacity due to their large volume; (v) with aging or damage they can ‘biodegrade’ via the apoptosis pathway; (vi) they are naturally tolerated by the body and display immune-biocompatibility from autogenous or immune-compatible donors and finally, (vii) they possess an inherent targeting ability [66, 67].

With atherosclerosis being a disease of the circulatory system, there is significant interest in investigating whole-cell drug delivery methods that utilize cell types such as RBCs, platelets, monocytes and neutrophils for its treatment [62, 66, 68]. The advantages of using these cells types in drug-delivery systems include the following: RBCs are able to prolong drug circulation time [69, 70]; platelets naturally secrete cytokines involved in haemostasis and tissue repair [71]; monocytes have the largest drug loading capacity and perform well when targeting ECs [68, 72]; and neutrophils have the potential for large-scale preparation and administration, and characteristically accumulate at an inflammatory site [36, 73].

To load therapeutic agents into RBCs, a hypotonic method is commonly used [19, 63, 66, 68]; this method allows retention of a large number of the cell’s biophysical and immune characteristics [63]. Claude and colleagues, however, have designed an elegant drug delivery system using artificial lipid vesicles [36], enabling drug delivery without destruction of the cell’s membrane structure. This method effectively prolongs the half-life and stability of the drug *in vivo*. Young’s team compared these methods and showed that using the latter method, the drug’s half-life *in vivo* was 4.5 ± 0.5 days, compared to just 2.4 ± 0.7 days using RBCs prepared using hypotonic treatment [74].

Encapsulating drugs in bioactive cells solves the problem of drug biocompatibility. But several disadvantages remain, including (i) strict *in vitro* storage conditions are necessary to ensure that cellular biological activity is maintained [19, 63, 66, 68] and (ii) drugs may be partially phagocytosed by the carrier cells. These factors limit the utility of the whole-cell carrier system [62], and, therefore, create the need for improved drug delivery methods.

## Extracellular vesicles as drug carriers

EVs, also called microparticles, are small vesicles released by most eukaryotic cells during processes such as activation and apoptosis [37, 75, 76]. EVs play a crucial role in intercellular communication, carrying biological materials such as cell membrane/plasma proteins and RNA, and enabling the cell to modify the phenotype and the function of target cells [77]. At present, there is no standardized classification of EVs, but they can be categorized by their parental cells, e.g. endothelial progenitor cell (EPC)-derived EVs, stem cell-derived EVs, neutrophil-derived EVs and platelet-based EVs. EVs can also be categorized by size (diameter) and biogenesis mechanism into four distinct classes: microvesicles (MVs, 100–1000 nm), apoptotic vesicles (>800 nm), exosomes (40–100 nm) and membrane particles (50–80 nm) [40, 78]. In this review, the term ‘EV’ mainly refers to MVs, exosomes and membrane particles.

In recent years, EVs have been used as carriers for nanomaterials and have demonstrated superiority compared to traditional drug delivery systems [79, 80]. As they are derived from cells, EVs possess an intrinsic biocompatibility and exhibit low cytotoxicity [81]. EVs also have the benefit of being able to evade immune elimination and complement activation [77].

The EV biomimetic loading systems not only preserve the physicochemical properties of therapeutic agents but also enhance the stability and targeting capabilities of the nanocarrier. Studies in cardiovascular disease have revealed that EVs released from cells such as platelets and neutrophils show a high targeting specificity for the inflammatory and tissue sites [82]. These observations demonstrate that the EV drug carrier system holds promise for the treatment of diseases such as atherosclerosis.

Peripheral arterial disease caused by leg atherosclerosis occlusion is an important manifestation of systemic atherosclerosis [83], and lack of proper blood perfusion to limbs can have serious consequences. Both the Ranghino *et al*. [84] and Deregibus *et al*. [85] laboratories showed that, in a mouse hind limb ischemia model, paracrine mediators released by EPCs participate in neovascularization following ischemia. In contrast with EPCs, EPC-derived EVs do not express HLA antigens, so EVs can be collected in large quantities and be delivered to ischemic patients irrespective of their HLA patterns. In addition, some researchers [86] pinpoint that stem cell-derived EVs can modulate the key processes of angiogenesis, including the proliferation, migration and formation of ECs, and promote the expression of angiogenesis-related genes and proteins [87–89]. This suggests that stem cell-derived EVs play a variety of roles to maintain endothelial integrity and prevent the development of vascular diseases.

It is relatively easy to obtain large quantities of EVs from neutrophils due to the number of circulating leukocytes. Gao’s team designed a nanovesicle-based drug delivery platform whose core consisted of cell membrane-formed nanovesicles generated from activated neutrophils [90]. They tested the effect of TPCA-1-loaded nanovesicles extracted from HL-60 cells and found that neutrophil-derived EVs reduced acute lung inflammation caused by lipopolysaccharide. Analysis of integrin β2 expression on neutrophil-derived EVs, which can bind ECs through ICAM-1 molecules [91], showed significantly higher numbers compared to neutrophils, indicating that these neutrophil-derived EVs have the ability to migrate to inflamed vasculature [90]. Further research from the same laboratory showed that piceatannol-loaded nitrogen cavitation neutrophil-derived EVs can reduce acute lung inflammation caused by lipopolysaccharide more than free piceatannol [92]. The two studies, among others, have demonstrated that drug-loaded neutrophil-derived EVs have potential utility in the treatment of inflammatory diseases [93, 94] and cancer [95].

Platelet-derived EVs are integral to thrombosis, and studies have shown that these EVs have 50–100 times the coagulant activity of activated platelets in blood circulation [96]. In one study, tail intravenous administration of EVs isolated from platelets exerted the ability to induce haemostasis in a tail snip bleeding model mice [97]. Compared with the control group, platelet therapy reduced bleeding by 45%, while platelet-derived EVs therapy reduced bleeding by 62%. Researchers also have shown that under the action of high shear stress, an increase in levels of platelet-derived EVs can enhance the adhesion of platelets and fibrin in the damaged atherosclerotic vessel wall, thus contributing to blood coagulation [98, 99].

The small size of EVs affords them the ability to penetrate deep into tissues, and they may even be able to cross the blood–brain barrier and access target sites that traditionally pose challenges for drug delivery [100]. However, despite the advances outlined in this article, there are several challenges still faced by this drug delivery method: (i) a consensus agreement regarding the gold-standard method for collection and isolation of EVs is still lacking; (ii) current research has not yet elucidated the molecular mechanisms involved in the biological effects of EVs; (iii) the effects of the introduced EVs on the delicate balance between the body and the effects of normal EVs on inflammation, coagulation, and vascular function are not fully understood and (iv) further *in vivo* studies investigating the potency and toxicology of EVs are necessary.

In summary, cell-derived vesicles have indeed shown strong application value in different diseases [101]. However, the current quality of production, isolation and purification methods limits their further clinical popularization [102]. Nevertheless, our growing knowledge about the mechanism of action of EVs and their potential use as therapeutic agents in various conditions provide exciting lines of investigation for the future.

## Cell membrane-camouflaged NPs

Cell membrane coating technology was first put forward by Zhang’s team in 2011, and it involves camouflaging NPs with entirely natural cell membranes. With the cell membrane coated directly, biomimetic NPs successfully transfer both membrane proteins and lipid bilayers while translocating natural cell membranes [13]. This enables cell membrane-coated NPs to take advantage of the nature cell’s surface antigen diversity. Studies have found that under the natural cell membrane camouflage, biomimetic NPs can be modified and functionalized by self-recognition [11]. They also demonstrate long-term blood circulation and can escape immune capture [11]. Therefore, cell membrane biomimetic NPs are being widely explored in the treatment of different diseases and are leading a new research direction.

### Synthesis of cell membrane-coated NPs

**Figure 2:**
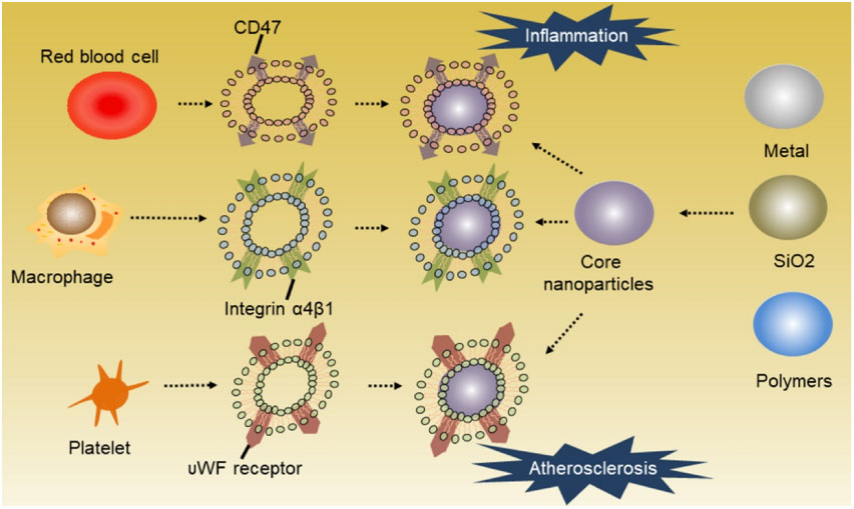
Cell membrane-coated NPs designed for atherosclerosis and inflammation therapy. The membranes of RBCs, platelets and macrophages were extracted and used to coat different kinds of NPs depending on the site of inflammation and atherosclerosis. Each cell membrane has its own unique surface proteins, such as CD47 on the RBC, integrin α4β1 on the macrophage and GPIIb/IIa on the platelet, modifying its therapeutic effect.

With the continuous development of biomimetic nanotechnology, the preparation methods of cell membrane camouflaged NPs have gradually improved and can now be summarized as a three-step process [103]: (i) cell membrane is isolated from the source cells (cells may be lysed by different methods, and then differential centrifugation is applied to separate cell membrane fragments); (ii) prepared membrane debris are repeatedly extruded from the polycarbonate membrane whose pore diameter is generally 200–400 nm, to obtain cell membrane microcapsules with proportionate particle size [103], and the core of drug-loaded NPs is prepared and (iii) NPs are encapsulated into cell membrane microcapsules. Source cells are usually derived from *in vitro* culture or whole blood isolation (especially for RBCs and platelets) (Fig. 2). The method used to purifying cell membranes and encapsulate NPs into membrane microcapsules may depend on the source cells used.

Many innovative approaches have been proposed to enable the use of RBC membranes, including hypotonic dilution, hypotonic dialysis, hypotonic preswelling, osmotic pulse method, chemical perturbation of the membrane and electrical breakdown [19]. In terms of covering NPs with the RBC membrane, the co-extrusion method is most commonly used, particularly in the targeted treatment of atherosclerosis. This involves RBC membranes vesicles and NPs being ‘squeezed’ together to form membrane-coated NPs.

On the other hand, the platelet membrane is generally obtained by a repeated freeze–thaw process [104]. According to Zhang’s research, they obtained platelet membrane through freezing aliquots of platelet suspensions at −80°C before thawing them to room temperature. To achieve membrane cloaking after purification, platelet membrane vesicles were dispersed and fused with PLGA particles by sonication at a frequency of 42 kHz and a power of 100 W for 2 min [105].

To synthesize macrophage-coated NPs, macrophages are collected from *in vitro* culture and resuspended in ice-cold *tris*-magnesium buffer. A mini-extruder, rather than a polycarbonate membrane, is then utilized to extrude macrophages and disrupt the cells. To isolate the cell membrane, centrifugation at 3000 *g* and 4°C for 30 min is necessary after pre-centrifugation. Co-extrusion with traditional NPs or other drug delivery systems through polycarbonate membrane at 200–400 pore size is proverbially employed for macrophage cell membrane cloaking [106].

### Red-blood-cell membrane-coated NPs

RBCs, or erythrocytes, are long-term circulatory delivery vehicles *in vivo* [16]. RBC membranes possess remarkable biocompatibility and non-immunogenicity properties, which contribute to its applicability in membrane-biomimetic NPs for atherosclerosis treatment [107]. Polysaccharides on the surface of RBC membranes are highly hydrophilic, which increases the stability of RBC membrane-coated NPs. Furthermore, the sustained release of small molecule drugs can be realized by their semipermeable membrane [108].

RBC membranes contain fibrinogen receptors [109]. Jo Seongjae’s team researched a sensitive fibrinogen biosensor NP coated by RBC membrane, which can be used for the quantitative determination of fibrinogen – one of the clinical diagnostic markers of cardiovascular disease in blood [110]. This biosensor has a broad fibrinogen determination range (0.001–5.000 mg/mL) and can detect high or low levels of fibrinogen in plasma. In addition, RBC membrane proteins can prolong NP cycle time in organisms while providing subtle immune escape abilities [111]. This feature effectively eliminates the disadvantage of poor biocompatibility of traditional NPs.

Piao and Gao jointly demonstrated that CD47 on RBCs protects gold particles from interacting with multiple compounds, and enables avoidance of phagocytosis by macrophages [107, 112]. Therefore, RBC membranes can greatly improve the controllable life of drugs *in vivo* and form a reliable drug delivery system.

Fan’s research group [113] has developed a coating scaffold with RBC membrane-coated NPs, which can eliminate the short-term inflammatory reaction induced by NPs, including neutrophil infiltration and significant increase of pro-inflammatory cytokines. The overlay of RBC membrane overcomes the unfavourable situation caused by polylactic-*co*-glycolic acid (PLGA) NPs, whereby the ratio of monocyte/macrophage decreased from 44.63 ± 1.65 to 12.7 ± 4.44%, and the number of recruited inflammatory cells decreased from 0.22 ± 0.12 to 0.14 ± 0.04 million per scaffold to the blank MA-alginate scaffold.

In 2019, Wang’s team designed a rapamycin-loaded PLGA-NP method coated by RBC membrane to effectively treat atherosclerosis [18]. Animal experimental results showed that the presence of the RBC membrane reduces macrophage-mediated phagocytosis in blood and enhances accumulation of NPs in atherosclerotic plaques, ultimately improving the drug effect.

Since 2011, when RBC membrane coating technology was designed by Zhang’s team for the first time [112, 114–119], it has been used in many systems. After modification by RBC membrane coating, the zeta potential of NPs decreases and the circulation time of NPs greatly increases [107, 112, 116–118]. Excellent immune evasion effectively prolongs the circulation time of RBC membrane nanocomposites, thus maintaining sufficient drug concentration in blood [120, 121]. This technology is now widely used in treating leukaemia, bacterial infections, chronic lead intoxication and atherosclerosis [122–124].

### Platelet membrane-coated NPs

Platelets are non-nuclear cells that originate from megakaryocytes [125]. They play a fundamental role in various physiological processes including cessation of bleeding, maintaining vascular stability and repairing damaged blood vessels [30]. Features of platelet membranes include immune escape [126, 127], intimal layer adhesion [128, 129] and the ability to interact with pathogens [130, 131].

**Figure 3:**
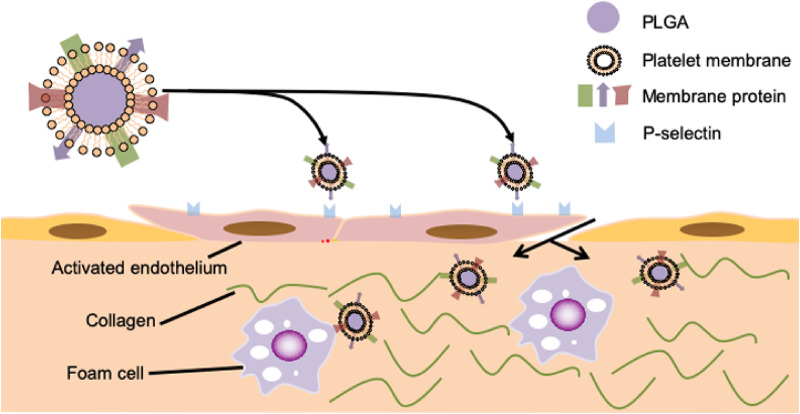
PNPs express surface markers that are capable of interacting with activated ECs, foam cells and collagen. This provides a theoretical basis for the design of platelet membrane-coated nanomaterials that actively target to atherosclerotic inflammatory sites.

Based on the physiological function of platelets and their characteristic adhesion and aggregation during the inflammatory process, it follows that using the platelet membrane as a coating material for NPs can effectively improve their targeting to atherosclerotic inflammatory sites [132]. The application of platelet membrane-coated NPs (PNPs) in atherosclerotic plaque imaging supports this notion [133]. Flow cytometry and fluorescent imaging *in vitro* have demonstrated that PNPs are capable of binding foam cells, collagen and activated ECs, which play a role in atherogenesis [134, 135], as shown in Fig. 3. Data from Oil Red O lipid staining ex vivo and *in vivo* provide evidence that PNPs are superior in targeting atherosclerotic plaques compared to PLGA NPs functionalized with polyethylene glycol or RBC membrane.

Owing to the excellent targeting of atherosclerotic plaques by platelet membrane biomimetic particles, this technology can also be applied in the treatment of atherosclerosis. Song’s team [136] designed platelet membrane-coated rapamycin PLGA NPs as a targeted drug delivery system for this purpose, and the presence of platelet membrane increased drug-loading capacity and encapsulation efficiency, while also improving the ability of rapamycin to induce macrophage autophagy.

Moreover, Hu *et al.* [105] studied the effect of platelet membrane-coated paclitaxel PLGA NPs in the experimental rat coronary artery restenosis model. The modification of platelet membrane improved the targeting ability of NPs to restenosis sites and significantly promoted the therapeutic effect of the drugs. However, due to the limited number and partial functionalization of proteins from platelet membrane in PNPs, it is impossible to generalize the accurate distribution and complexity of different glycoproteins on platelet membrane [35]. This results in short cycle time and poor targeting of PNPs compared with normal platelets, but their efficacy is still ∼7–8 times better than that of naked NPs [137].

### Macrophage membrane-coated NPs

Macrophages are derived from monocytes and are one of the innate immune cells involved in atherosclerosis [138]. They have numerous biological functions, such as antigen presentation, phagocytosis of bacteria and participation in inflammatory reactions [139]. In atherosclerosis, macrophages accumulate in lesion areas; a fact that provides theoretical support for a biomimetic nano-drug delivery system coated by the macrophage membrane.

At present, macrophage membrane-coated drug delivery systems are being applied in the field of atherosclerosis treatment. Li’s group successfully designed a new type of macrophage membrane-coated PLGA NP (MPL-NP) with Cao’s method [140]. The consequences of Western Blotting showed that functional proteins, such as integrin α4β1, remained on the surface of MPL-NPs and could specifically recognise atherosclerotic VCAM-1. This provided an opportunity for the nanocarriers to realise high accumulation at atherosclerotic lesion sites [34]. Results in animal models further demonstrated that the MPL-NPs possessed improved targeting to apolipoprotein E-deficient mice lesions versus ordinary NPs. The fluorescence intensity of MPL-NPs was 5.07 times that of PLGA NPs, and the signal from MPL-NPs partly overlapped with that of VCAM-1, indicating that they accumulated together in plaques [140]. In summary, the specific interaction between integrin α4β1 and VCAM-1 on the surface of macrophage membrane realizes excellent targeting of macrophage membrane-coated NPs to atherosclerotic lesion sites, and paves the way for a new method to transport drugs to lesion sites in atherosclerosis.

## Summary and prospect

NP-based drug delivery systems have utility in the treatment of atherosclerotic plaques. The advantages to these systems are that they can release drugs locally and they have a level of targeting ability. However, their short cycle time and low biocompatibility limit their therapeutic potential. In comparison, whole-cell-, cell membrane- or EV-based systems incorporate endogenous features that have various biological functions and contribute to better biocompatibility.

The cell-derived biomimetic drug delivery system elegantly combines the advantages of synthetic NPs and a natural bio-membrane, providing enhanced convenience and greater potential in the treatment of atherosclerosis. The advantages of this combined system include that it provides more options for the design of specific nano-drugs, and that the coating of cell membrane affords the nano-drug-loaded particles better biocompatibility, long circulation time and inflammatory site targeting.

Although some excellent achievements have been made in this field, there are many challenges that still need to be overcome before the biomimetic nano-drug delivery systems are applied to atherosclerosis treatment in the clinic. For example, the RBC membrane nano-drug delivery system displays a long circulation time [107], but it is not able to specifically target the drug to the site of atherosclerosis. In addition, the preparation of biomimetic NPs with a cell membrane is quite complex, and requires a higher technical ability than the preparation of ordinary synthesized NPs. In the large-scale production of biomimetic NPs, we still need to solve the problem of ensuring the integrity of cell membrane structure and the function of surface biological factors during the processes of extraction and coating of the cell membrane. Furthermore, shedding of cell membrane structure or the early release of drugs in circulation will cause a waning therapeutic effect.

For delivery systems using whole cells, the challenge still lies in the potential genetic risks, limited drug dosage, dysfunction in cell plasticity and mechanical stability. Moreover, EV-based delivery systems are associated with high cost and low recovery rate as well as deficiency of sufficient theoretical basis.

In the future, there is the potential for stem cell membranes, shedding vesicles and even the outer membranes of some bacteria to be used in the preparation of biomimetic nanomaterials for the treatment of atherosclerosis. There have been reports that RBC-derived MVs have been applied to photothermal agents to achieve anti-tumour effects. It is possible that some anti-tumour biomimetic nanomaterials may be useful in the design of nanomaterials for the treatment of atherosclerosis. With further research, there is no denying that the cell-derived biomimetic drug delivery system will push the targeted treatment of atherosclerosis to new heights.

## Funding

This study was supported in part by grants from the Open Fund for Key Laboratory of Biorheological Science and Technology, Ministry of Education (CQKLBST-2019-010), The Project of Tutorial System of Medical Undergraduate in Lab Teaching & Management Center in Chongqing Medical University (LTMCMTS201905) and Undergraduate Scientific Research and Innovation Laboratory Project of Chongqing Medical University (201965). The support from the Chongqing Engineering Laboratory in Vascular Implants, the Public Experiment Centre of State Bioindustrial Base (Chongqing) and the Laboratory of Tissue and Cell Biology are gratefully acknowledged.

*Conflict of interest statement*. None declared.
